# Robotic Workstations in Microextraction Techniques: Technological Evolution and Analytical Impact

**DOI:** 10.1021/acs.analchem.6c02053

**Published:** 2026-06-23

**Authors:** Guillem Peris-Pastor, Juan L. Benedé, Alberto Chisvert

**Affiliations:** GICAPC Research Group, Department of Analytical Chemistry, 16781 University of Valencia, Burjassot, Valencia 46100, Spain

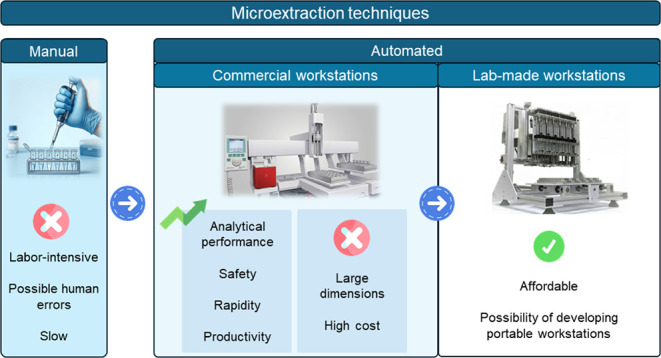

## Introduction

Current trends in Analytical Chemistry include a constant effort to achieve sustainable methods,^1^ not only looking for low (or even negligible) environmental impact but also saving time, money, energy, and resources, and, of course, solving the analytical problem, as demanded by the White Analytical Chemistry^2^ and the Sustainable Development Goals.^3^ Most specifically, in the sample preparation field, traditional extraction techniques, such as liquid–liquid extraction (LLE) and solid-phase extraction (SPE), have been progressively miniaturized to the so-called microextraction techniques,^4^ where the high amounts of consumed organic solvents are considerably reduced,^5,6^ thus also complying with the principles of Green Sample Preparation.^7^ Despite their many advantages, however, microextraction techniques often still involve manual operations that might be time-consuming, labor-intensive, and/or prone to human error, which limits their practicality in high-demand applications.^8^

Automation offers a powerful solution to these challenges as it aims to obtain quicker, safer, and more accurate procedures compared to manual ones.^9^ On one hand, automated systems are able to perform operational tasks faster than humans can do, and some of them are able to operate over extended periods, including overnight runs, thus increasing productivity.^10,11^ This feature is especially relevant in high-throughput settings or when a large number of samples need to be processed within tight deadlines. On the other hand, although it is true that toxic chemicals have been progressively replaced by less toxic ones^12^ and their amounts have been reduced,^13^ automation prevents operator’s exposure to these (more or less) toxic chemicals, thus enhancing the safety of the procedure. Finally, even though analytical procedures are usually carried out by expert professionals, unintentional human errors related to different operational tasks can occur, thus leading to imprecise and inaccurate results of the analysis. Automating the operational tasks minimizes the human intervention and thus these unintentional errors, guaranteeing better analytical results.^11^

Automation also provides additional economic benefits, since as faster procedures are obtained, more samples per unit of time can be analyzed. Furthermore, the employment of automated systems is also related to fewer expenses in personnel who will not need to be as highly qualified.

All these advantages were echoed in the sample preparation field to the point that automation has been included as one of the main criteria of the Green Sample Preparation principles,^7^ which were proposed in order to be a guideline for researchers to develop more sustainable methods. In fact, several green and sustainable metric tools include automation as one of their evaluated criteria.^14,15^

Different approaches for LLE and/or SPE based on classical flow techniques (i.e., flow (FIA) and sequential (SIA) injection analysis)^16^ and on more recent multisyringe and microfluidic systems,^17–19^ passing through column-switching methods,^20^ have been extensively reported. The use of robots, although less so, has also been applied to LLE and SPE.^21^ At this point, it should be mentioned the well-known Symbiosis system (formerly Prospekt), developed by Spark Holland, which integrates a column-switching approach to couple SPE with liquid chromatography (LC) along with robotic arms for aspirating samples and automatically exchanging SPE cartridges (https://www.youtube.com/watch?v=asdm6Onwq0Q). However, despite the first robots for multistep sample handling being introduced in the early 80s,^22,23^ widespread adoption of dedicated commercially available robots in sample preparation continues to be limited due to their high cost and, as such, remains inaccessible to many laboratories.

The achievements in automating microextraction techniques have been less reported, which is mostly attributed to the experimental workflow required by some of them.^24^ In this regard, it should be emphasized that the use of robots, although constituting an expensive solution, could provide the needed workflow. Fortunately, the recent emergence and spread of open-source microcontrollers governing low-cost gadgets^25,26^ and the easy accessibility of 3D printing^27^ enable nonspecialist researchers in electronics and programming to design and construct workstations as alternatives to costly commercial solutions.

Recently, several descriptive review articles related to this topic have been published,^8–11^ but none of them specifically focus on the automation of microextraction techniques using robotic workstations and their evolution over time. In this sense, the aim of this review is to describe, in a comprehensive and critical way, the technological evolution in terms of automation by using robotic workstations of the most widely used microextraction techniques since they emerged, offering an overview of the most notable advances in the field related to the automation itself. Special emphasis has been placed on multiextraction platforms (e.g., use of 96-well plates) and lab-made workstations, as well as on those nonautomated approaches that present a high potential for automation but have not been automated due to lack of resources. Please note that applications of previously developed approaches to other analytes and/or matrices are not discussed, nor are similar articles using innovative sorbent materials, unless they cause an improvement in the automation level. A general comparison of automated robotic systems with manual ones in terms of throughput (i.e., productivity), intervention of the operator, and other relevant aspects like the economy and portability of the workstation is also discussed to highlight their analytical impact.

## Automation of Sorbent-Based Microextraction Techniques

### Micro-Solid-Phase Extraction

Micro-solid-phase extraction (μSPE) is considered the natural miniaturization of classical SPE. It follows exactly the same principles as SPE^6^ of flowing sample and involves solvents through a sorbent confined in a cartridge or in a disk by applying vacuum from the lower part or positive pressure on the upper part. It could be considered as the result of progressively reducing the amount of sorbent as a consequence of having smaller sample quantities and/or reducing the amounts of solvents for environmental reasons. Due to the workflow involved in the conditioning, loading, washing, and elution steps by flowing liquids through the sorbent, its automation was rapidly accomplished in the early 90s with the popularization and availability of commercial robotic workstations equipped with multichannel liquid handling arms that operate over (usually) 96-well μSPE plates. Currently, different alternatives from various manufacturers offering their technology are readily available, ranging from single-channel workstations to the more common 8- or 12-channel systems, or even up to 96 channels, for loading samples/solvents onto the 96-well μSPE plate. Regardless of the number of channels on the arm, once the 96 samples are loaded, they are subsequently subjected to μSPE in parallel. To review all these robotic workstations is a huge task that surpasses the aim of this article. As an example, the contribution of Antonelli and coworkers,^28^ who employed a Tecan Freedom Evo 150 robotic system, should be mentioned. It was composed of an 8-channel liquid handling arm, surrounded by reservoirs containing the different involved solvents (i.e., conditioning, washing, and elution ones) and by 96-well plates containing samples and μSPE devices mounted on plate shakers to which vacuum was applied, and by a 96-well plate-handling arm to move these plates (its operational workflow can be briefly envisioned at https://www.youtube.com/watch?v=6z4lc_26duk). Once the extraction procedure is finished, the 96-well plate containing the extract is covered manually with a cap tap and transferred to the chromatographic autosampler. Even though 96-well plate platforms provide a high-throughput analysis, Jagadeesan and coworkers improved this aspect using 384-well plates.^29^ These authors employed a Biomek 3000 robotic workstation, which works similarly but applies positive pressure instead of vacuum as the driving force.

However, it should be mentioned that vacuum or positive pressure control, and therefore flow homogeneity through the 96 (or 384) wells, is a challenge due to possible plugging, channeling, or uneven well permeability, resulting in some wells drying up before others drain, which could jeopardize the results.^30^ This could be solved with other sorbent-based microextraction approaches discussed in the following sections.

### Pipette-Tip Solid-Phase Extraction

Pipette-tip solid-phase extraction (PT-SPE) is a type of μSPE that emerged in 1998, where a very small amount of sorbent is packed between two frits in the bottom part of a pipette tip, which is conveniently assembled to a pipettor.^31^ Leaving aside the conditioning step, carried out similarly but with the conditioning solvent, the extraction process starts by aspirating the sample solution from the sample flask into the tip and then dispensing it back into the sample flask. This aspirating/dispensing cycle can be replicated as many times as needed, thus increasing extraction efficiency compared to conventional μSPE. Then, after aspirating/dispensing the washing solvent, the analytes are eluted by aspirating/dispensing the elution solvent. The operational workflow allows it to be readily automated by means of commercially available multichannel pipetting workstations, and many applications can be found in the analytical literature using this technology. It is worthy to mention the contribution of Abdel-Rehim and coworkers,^32^ who used a commercially available robot handling a 96-sorbent-packed pipette tip array able to process 96 samples simultaneously. The extraction procedure was composed of the following steps. First, samples were loaded, respectively, in the wells of a conventional 96-well plate, whereas the 96 tips were taken by the robotic arm. Then, all samples were aspirated/dispensed simultaneously, thus passing in parallel through the corresponding pipette tip. After that, each sorbent contained in each pipette tip was washed by aspirating/dispensing the washing solvent, and subsequently, the target compounds were desorbed over a new 96-well plate by aspirating/dispensing the desorption solvent for further analysis.

Nevertheless, the main reported drawbacks for PT-SPE are related to plugging and thus overpressure problems,^33^ which could be alleviated by using monoliths, paper-based enrolled sorbents, or functionalized inner-wall pipette tips.^34^ The dispersive (or disposable) pipette extraction (DPX) approach described later constitutes an efficient pipette-tip-based alternative to PT-SPE.

### Microextraction by Packed Sorbent

Microextraction by packed syringe, or microextraction by packed sorbent (MEPS) as currently known,^35^ emerged in 2004 as another type of μSPE, where the sorbent is packed inside a gastight glass microsyringe barrel or between the barrel and the needle as a cartridge. Like in PT-SPE just above commented, the solutions/solvents are flowed in two directions (up/down), in this case by pulling out/compressing the microsyringe plunger. The sample is aspirated for one or several cycles, retaining the analytes; then the sorbent is washed (if needed), and finally, taking advantage of the microsyringe-based design, the analytes can be desorbed, unlike PT-SPE, directly into the injection port of the instrument by flowing the elution solvent. Since the entire workflow is carried out in a microsyringe, this approach is fully compatible with commercially available autosamplers. In fact, in the first attempt, MEPS was already automated, employing a CombiPAL autosampler for gas chromatography (GC), where the microsyringe was exchanged and the sample/solvent vials were placed in the autosampler tray.^36^ In this way, the sequence of the autosampler is launched, allowing the automated extraction and further instrumental analysis of the samples one by one. Furthermore, after each extraction and subsequent injection, the microsyringe extraction device is automatically washed to avoid carryover in the following extraction. Different automated MEPS methods using autosamplers from different manufacturers can be found in the analytical literature.^37^

At this point it should be mentioned the lab-made workstation developed by Lanças and Santos-Neto’s research group^38^ to carry out MEPS in an automated way (see Figure 1a). It was constructed with low-cost gadgets and controlled by the open-source software Arduino. This workstation mainly consisted of a Cartesian robotic arm with an inverted microsyringe, similar to a chromatography autosampler, and a horizontal plate holding a rack of vials on stirrers. This plate is moved in *z*-axis to place a row of vials below the arm’s line of action, which is allowed to move in *x*-axis to find the selected vial from this row, and in *y*-axis to access the vial. Furthermore, the microsyringe driver can also be equipped with a three-way solenoid valve in order to integrate the sample preparation workstation with an LC system in such a way that in the “off” position the syringe barrel is connected to the needle, whereas in the “on” position it is connected to the LC injection loop. Initially, with the solenoid valve in the “off” position, the microsyringe works to conduct MEPS. After extraction, the valve commutes to the “on” position, and the enriched extract contained in the microsyringe barrel is ejected into the LC sampling loop. Later, this workstation was improved to carry out up to six extractions in parallel (Figure 1b).^39^ In this case, it was equipped with six inverted microsyringes linearly aligned on a plate to hold 6 × 8 conveniently arranged vials, some of them containing the samples and solvents, and others were empty to collect the extracts. Through different stepper motors controlled by Arduino, the plate was moved horizontally to locate the appropriate vials below the microsyringes, which were moved up and down to enter/release the vial, and then their plungers were moved to aspirate/eject the solutions.

**Figure 1:**
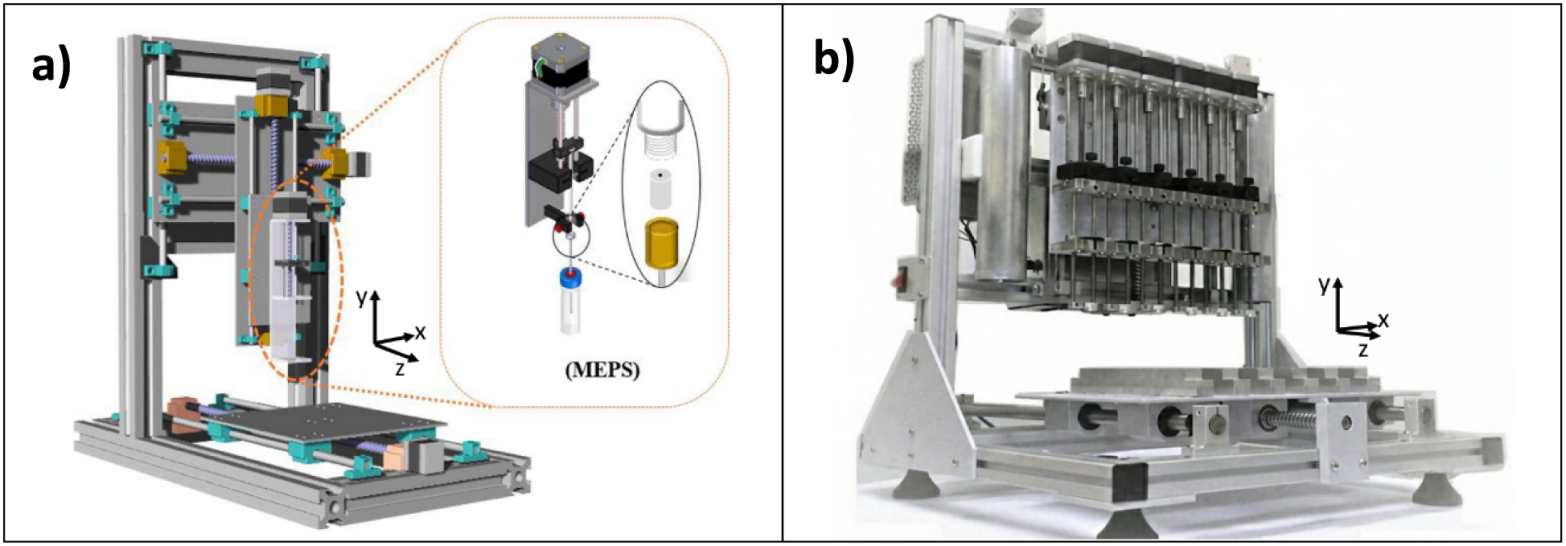
Lab-made workstation developed by Lanças and Santos-Neto’s research group for: a) MEPS, adapted with permission from ref 38. Copyright 2019 Elsevier; b) parallel MEPS, adapted with permission from ref 39. Copyright 2020 Elsevier.

### Solid-Phase Microextraction

Solid-phase microextraction (SPME) was presented by Pawliszyn and coworkers in 1989 ^40^ and soon became a cutting-edge technique in the field of sample preparation. Unlike μSPE, neither is the sorbent confined nor do the sample and solvents flow through the sorbent, but the sorbent is chemically coating a thin fused silica (or metallic) fiber, which is directly immersed (DI) in a liquid sample or exposed to its headspace (HS). After stirring for a defined period of time, it is subjected to desorption for its subsequent measurement in an analytical instrument. In a first attempt, fiber was directly inserted into an injection port of a GC instrument after extraction for thermal desorption. A metal sheath piercing the septum was used to guide the fiber, thus avoiding its breakage. Later, this same research group headed by Prof. Pawlisyn improved the operational workflow by proposing a microsyringe where the metal plunger wire assembly was replaced with the fiber and whose end was pulled out or withdrawn through the tip of the needle, which served to pierce the septum of the extraction vial or of the GC injection port.^41^ This led to a purpose-designed device made and commercialized by Supelco,^42^ and it continues to be the most widely used workflow of this technique. Supelco also introduced a desorption interface for LC instruments based on a first proposal, which was improved later.^43^ Alternatively, after the extraction, the fiber can be immersed in a vial containing a solvent to perform the liquid desorption of the analytes and subsequent injection into the GC, LC, or any other analytical instrument.

The first attempt at SPME automation was presented precisely by its inventors in 1992,^44^ even before the development of the commercialized devices mentioned above.^45^ In this approach, the SPME fiber was attached to a stainless steel tubing, which was inserted into a microsyringe plunger. This device replaced the microsyringe of a commercial GC autosampler, taking advantage of the fact that the SPME workflow is analogous to that of a conventional microsyringe. Thus, in principle, any GC autosampler could be used to automate SPME prior to GC. The major challenge was incorporating agitation and temperature control. In this regard, a magnetic stirrer in a vertical position was placed at the location of the washing vial. Extraction vials were prepared by manually adding samples and stir bars and left in the tray. After that, the tray, which had the ability to move forward and backward, was moved in order to set the first vial under the SPME fiber and 3 cm away from the stirrer, which was always left on, so that the sample to be extracted was under agitation. Then, the fiber was inserted into the vial at its desired length. After the extraction was performed, the fiber was automatically transferred to the GC injector at the chosen depth to carry out the thermal desorption of the analytes. Even though the extraction procedure itself was entirely automated, the autosampler was not able to control the temperature of each vial, thus preventing the performance of HS-SPME or controlling the temperature of the sample in DI-SPME (if needed).

Some years later, instrument manufacturers, in collaboration with SPME inventors, launched GC autosamplers adapted to carry out SPME in a more efficient way. The improvement included vibration of the fiber or rotation of an agitator tray for stirring the sample, thus avoiding the manual introduction of the stir bar and its subsequent cleaning procedure, and a thermostated sample tray.^46^ Figure 2a shows an SPME module integrated in a commercially available CombiPAL autosampler. Later and similarly, the same authors used a Twin PAL dual-arm robotic system to increase the automation level.^47^ One of the arms consisted of a syringe to handle liquids to prepare standards, carry out dilutions, and add reagents to the vial containing the sample, whereas the second arm consisted of an SPME sampling. This system also had a magnetic vial transporting platform to move the vials to the desired place and other modules for heating/cooling and stirring.

**Figure 2:**
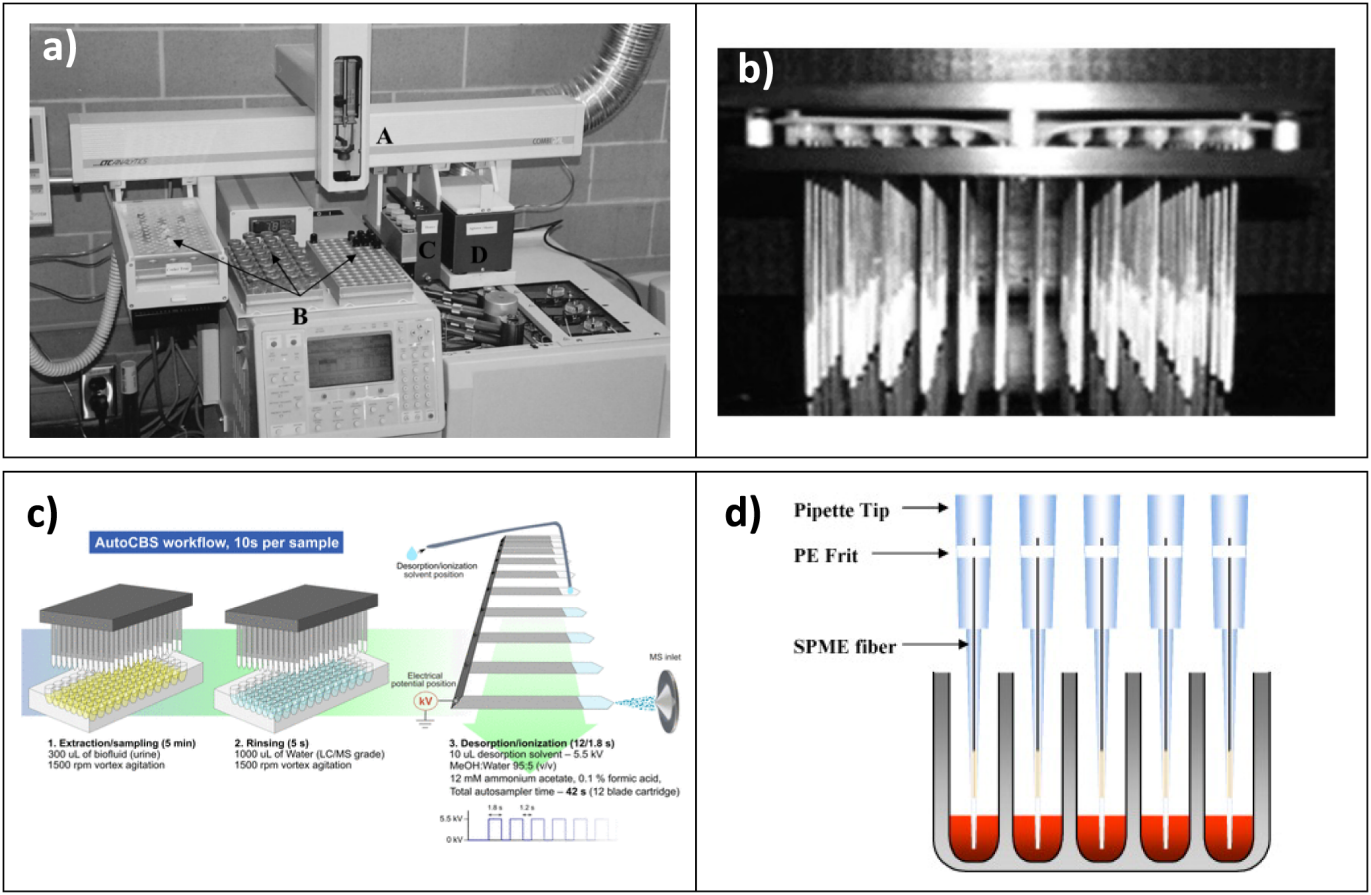
a) CombiPAL autosampler provided with an SPME module composed of: (A) sample preparation/injection arm, (B) sample tray, (C) needle heater, and (D) heater/agitator. Reproduced with permission from ref 45. Copyright 2005 John Wiley and Sons. b) Custom-made 96-multifiber SPME device developed by Pawliszyn’s research group. Adapted with permission from ref 49. Copyright 2008 American Chemical Society. c) Experimental setup for extraction, desorption, and ionization of analytes via automated CBS. Reproduced with permission from ref 53. Copyright 2019 American Chemical Society. d) SPME fibers embedded into pipette tips for 96-well plate liquid handlers. Adapted with permission from ref 54. Copyright 2009 Elsevier.

In order to increase the throughput of SPME, Pawliszyn’s group constructed an array of 96 stainless steel pins covered with silicone hollow fiber membranes acting as SPME devices, which were strategically positioned to fit in the centers of each well of a commercially available 96-well plate.^48^ First, they used it manually, and later they automated it with the aid of a three-arm robotic system from Professional Analytical System (PAS) Technology.^49^ The first arm was used to hold, transport, and position the custom-made 96-multifiber SPME device (see Figure 2b) for the extraction and liquid desorption steps of SPME; the second arm was equipped with a N_2_ blow-down device to perform solvent evaporation (if needed); and the third one was equipped with a syringe to dispense reconstitution/desorption solvents or internal standard solutions into the individual wells, as well as to perform sample injection into the injection port of an LC instrument. Orbital shaking was employed as the stirring method during the extraction and desorption steps.

The same research group later expanded this technology to blade SPME with thin-film geometry (known as thin-film microextraction (TFME)), thus enhancing extraction efficiency due to the higher extraction area and sorbent amount.^50^ Later, they automated what they previously coined as coated blade spray (CBS).^51^ In CBS, the blade SPME devices were coupled to ambient mass spectrometry (AMS), where extracted compounds were directly desorbed and ionized by forming a spray after applying a drop of desorption solvent and a high voltage between the blade and the entrance of the mass spectrometry instrument. These authors used the 96-well plate containing 96 SPME blades to extract the target compounds by using the automatic system previously described, and then, the blades were manually disassembled and installed on the interface for AMS analysis.^52^ In a subsequent improvement, they constructed an autosampler that allowed loading a comb-like row with 12 blades, which was moved by a stepper motor to deliver blades sequentially in front of the AMS entrance, where desorption solution was dropped by a pump and the electric voltage was applied (see Figure 2c).^53^

Simultaneously, and with the aim of achieving compatibility between the original SPME fiber format and liquid handlers for 96-well plates, this same group developed a new approach based on embedding SPME fibers in pipette tips (coined in-tip SPME) (see Figure 2d).^54^ With this approach, samples/solvents were conveniently aspirated (as many times as required) by using the liquid handler of a commercially available Tomtec Quadra 96 workstation, thus getting in touch with the fiber to carry out the extraction, washing, and elution steps, respectively.

On the other hand, it should be mentioned here again that the above-described Cartesian robot developed by Lanças and Santos-Neto’s research group,^38^ which was conveniently configured, also allowed to carry out SPME in an automated way^55^ (see Figure 3a). The extraction device consisted of a homemade SPME sleeve assembled to the microsyringe needle. Initially, the three-way solenoid valve located in the head of the microsyringe barrel was in the “off” position, and the platform moved to place the extraction vial (with a magnetic stir bar inside) below the arm, which immersed the SPME device into the sample. After extraction, the arm took out the SPME device, and the platform moved to place the desorption solvent vial (or washing solvent vial, if needed) below the arm, which immersed the SPME device to desorb the analytes. Then, the enriched extract was aspirated into the syringe barrel, the valve was commuted to the “on” position, and the extract was ejected into the LC sampling loop.

**Figure 3:**
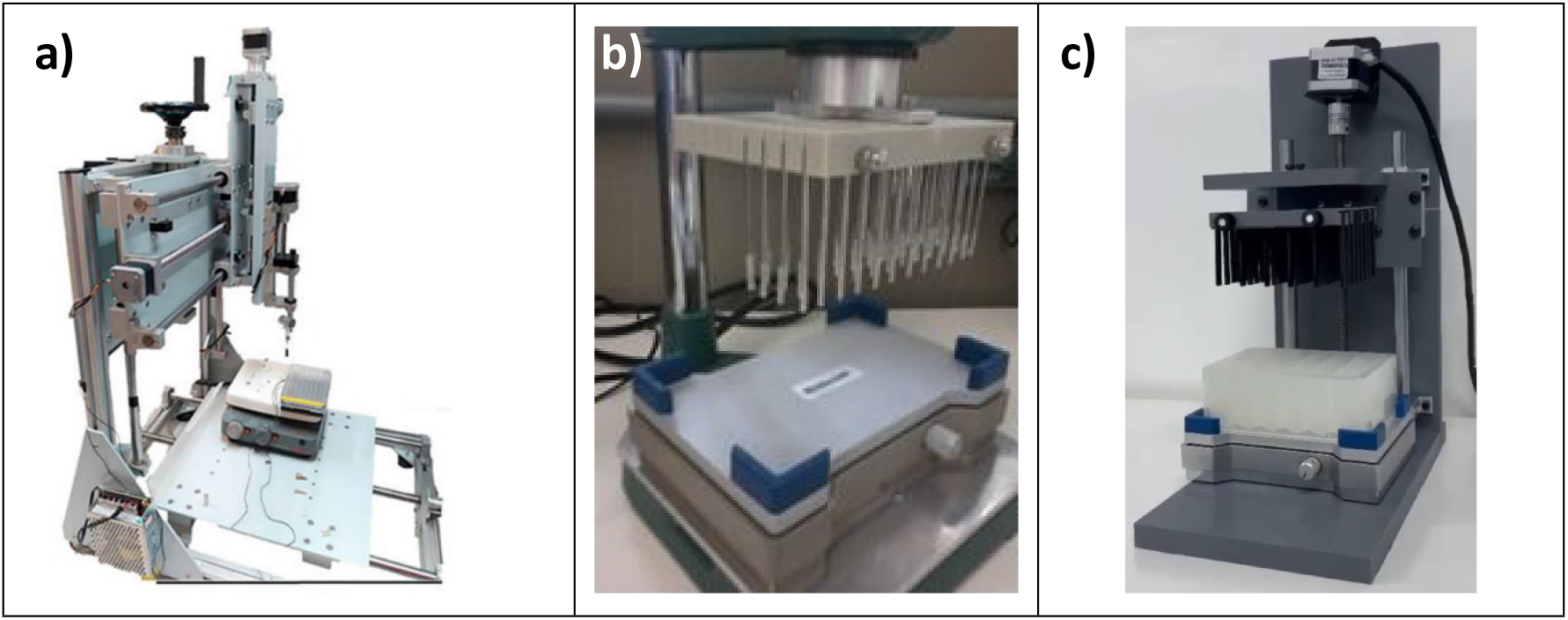
a) The lab-made workstation developed by Lanças and Santos-Neto’s research group for SPME. Adapted with permission from ref 55. Copyright 2021 Elsevier. b) The lab-made workstation developed by Carasek’s research group for TFME. Adapted with permission from ref 56. Copyright 2017 Elsevier. c) The lab-made workstation developed by Merib and coworkers for PANDA. Adapted with permission from ref 57. Copyright 2025 Elsevier.

Despite it not being an automated platform, it should be mentioned here the lab-made proposal from Carasek’s research group to perform TFME.^56^ They designed a platform with an array of 96 extraction blades by conveniently fixing eight comb-like stainless steel pins on a plastic plate; thus, they perfectly matched on a 96-well plate placed on an orbital shaker. They used a metallic frame that allowed lowering and raising the extraction plate by means of a manually operated lever (see Figure 3b). For each of the involved steps during the extraction, the 96-well plates were conveniently replaced manually.

More recently, Merib and coworkers developed an Arduino-controlled workstation to carry out a variant of SPME known as polyamide noncoated adsorption-based microextraction (PANDA),^57^ which consists of using 3D-printed polyamide blades as extraction devices. They fabricated a 6 × 8 pins array, which was mounted on a 3D-printed polylactic acid structure that allowed its up-and-down movement by using a metallic axis and a stepper motor (see Figure 3c). The samples to extract were placed in a standard 48-well plate located on an orbital shaker platform. To begin with the extraction process, the pin array was lowered and soaked into the 48-well plate, and then it was stirred. After the extraction was accomplished, the pin array was retired, and the 48-well plate was manually changed by another one containing the washing and/or elution solvent and finally transferred to the analytical instrument.

### Dispersive Solid-Phase Extraction

Dispersive solid-phase extraction (DSPE) was presented in 2003 by Anastassiades et al.^58^ This technique consists of the dispersion of a sorbent material within the solution of the sample to extract the analytes. Then, the sorbent was collected by centrifugation, thus discarding the supernatant. Subsequently, a desorption solvent (or a washing solvent, if needed) is added, and the sorbent is dispersed again to desorb the target compounds. Finally, the sorbent is separated by centrifugation, and the liquid extract is transferred to the analytical instrument. In the case of using magnetic sorbents, the paramagnetic properties of these materials allow for effective handling by applying an external magnetic field, simply provided by a magnet, thus avoiding centrifugation operations.^59^

Due to its operational workflow, the achievements in automating this approach are very scarce. The first attempt at a robotic workstation to automate miniaturized DSPE, i.e., dispersive microsolid-phase extraction (DMSPE), was that proposed in 2014 by Lee’s research group based on a CombiPAL commercially available autosampler.^60^ In this approach, a vial containing a suspension of a magnetic layered double hydroxide as sorbent was transferred by the autosampler to an agitator to ensure its homogeneity. After that, the desired volume of this suspension was withdrawn into the syringe and ejected into the sample vial, which was transferred to the agitator to disperse the sorbent material. After extraction, the vial was transferred to an autosampler tray with a prepositioned magnet to retrieve the magnetic material at the bottom of the vial. Then, the syringe removed and discarded the remaining donor solution in several cycles due to the limited syringe volume. After rinsing, the syringe added an acidic solution from a reagent vial to dissolve the magnetic layered double hydroxide and to release the analytes by agitating and controlling the temperature. Finally, an aliquot of the liquid extract was withdrawn by the syringe autosampler, and it was directly injected into an LC instrument.

A fully automated DMSPE method was presented also based on a commercially available mechanical arm that manipulates a magnetic sorbent by means of a magnetic rod surrounded by a plastic sleeve, which is submerged into different holes of a 96-well plate.^61^ The operational workflow is the following: first, the mechanical arm submerges the magnetic rod with the sleeve in a hole containing a dispersion of the magnetic sorbent, thus attracting the sorbent on the surface of the sleeve; then, the mechanical arm transfers the magnetic rod with the sleeve and sorbent material to another hole containing the sample, where the sorbent is dispersed after removing the magnetic rod and oscillating the sleeve; after extraction, the mechanical arm places the magnetic rod in the sleeve again to retrieve the sorbent; the procedure is repeated in a third hole also containing the sample solution; then, the sorbent material is washed in a fourth hole, and subsequently it is dispersed in the desorption solvent placed in a fifth hole to desorb the analytes; and finally, the magnetic material is retrieved similarly to leave the extract ready for analysis.

More recently, our research group presented a more austere lab-made robotic workstation compatible with low-volume samples to carry out DMSPE in a semiautomated way.^62^ This workstation was built employing low-cost electronic gadgets conveniently assembled in a 3D-printed casing (Figure 4a) and powered by an external battery of 9 V, which conferred it with total portability. First, the magnetic sorbent material was weighed into the extraction vessel and placed into the 3D-printed holder assembled to the electromagnet. Next, a volume of the sample was added, and an arm moved by a servomotor placed a bar-shaped magnet (attached to a DC motor) at a very short distance over the sample. Then, the DC motor was turned on, which rotated the magnet at high speed, thus dispersing the magnetic sorbent material in the liquid sample. After extraction, the DC motor and the electromagnet were simultaneously turned off and on, respectively, so the magnetic sorbent was sedimented and fixed, whereas the arm was drawn back to allow the operator to remove the remaining donor phase with a micropipette. This procedure was repeated with the washing solvent (if needed) and with the desorption solvent. Finally, the liquid extract was manually taken and transferred to a measuring instrument. This workstation executed the orders automatically by means of a microcontroller programmed in the C++ language, employing the open-source software Arduino. However, the addition and removal of liquids were performed manually.

**Figure 4:**
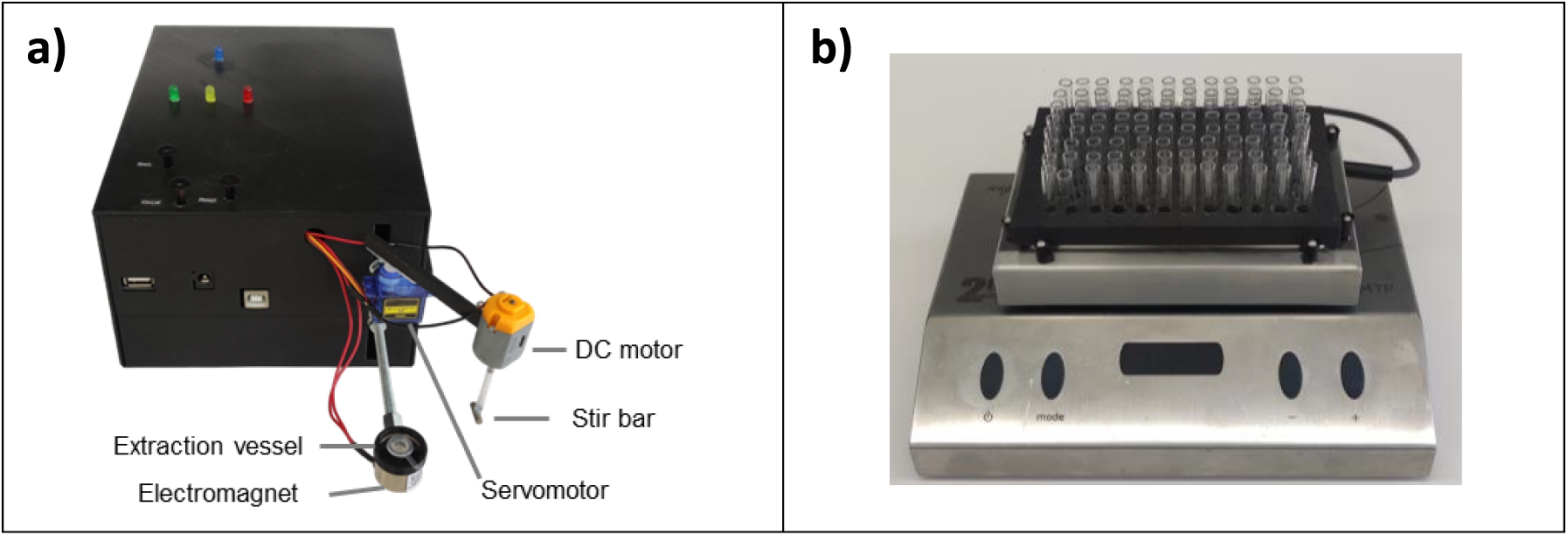
a) The lab-made robotic workstation developed by Chisvert’s research group for DMSPE. Adapted with permission from ref 62. Copyright 2025 Elsevier. b) The 96-position platform developed by Chisvert’s research group for DMSPE.

Aware that it is not an automated platform, we would like to include here our recently developed 96-position platform for DMSPE,^63^ which, conveniently adapted to a commercially available liquid handler workstation to add and to remove the involved liquids, could provide a very high-throughput platform to conduct DMSPE. This platform consists of a custom-designed 3D-printed support to accommodate 96 400-μL flat-base glass inserts as miniaturized extraction vessels containing the solutions to be extracted and cylindrical neodymium magnets, which are located on a 96-position magnetic stirrer (see Figure 4b). The extraction vessels are manually loaded to conduct the extraction step, followed by the washing and elution steps, which could be carried out by an appropriate multichannel liquid handler.

### Dispersive Pipette Extraction

The so-called dispersive (or disposable) pipette extraction (DPX), patented by Brewer in 2003,^64^ is a smart approach for carrying out DMSPE. DPX uses pipette tips as extraction devices connected usually to a micropipettor acting as the driving force, although they can also be connected to luer cone-type plastic syringes.^65,66^ Unlike PT-SPE described above, in DPX, the pipette tip contains the sorbent placed loosely between two frits in such a way that when the sample is aspirated, the sorbent is dispersed within the sample. Then, this dispersion is ejected, thus removing the liquid while retaining the sorbent in the lower frit. After that, the desorption solvent (or washing solvent, if needed) is aspirated, achieving a new dispersion to desorb the target compounds, and finally, it is ejected to get a liquid extract containing the target compounds. DPX has been readily automated by using commercially available liquid handling platforms from different manufacturers, and different applications can be found in the analytical literature, either carrying out a single extraction^67^ or even processing an entire 96-well plate in parallel.^68^ Just as an example, its operational workflow can be envisioned at https://www.youtube.com/watch?v=l-3cmKjYmYI.

Carasek and coworkers have recently developed a lab-made platform to conduct DPX that can process even up to six samples in parallel (see Figure 5a).^69^ This platform accommodates six linearly aligned luer-lock syringes provided with six pipette tips hanging above a plate containing the sample and solvent vials. Different stepper motors controlled by Arduino move vertically the syringes and their plungers to access and to aspirate/eject the solutions, whereas another stepper motor moves the plate horizontally to place the appropriate vials below the syringes.

**Figure 5:**
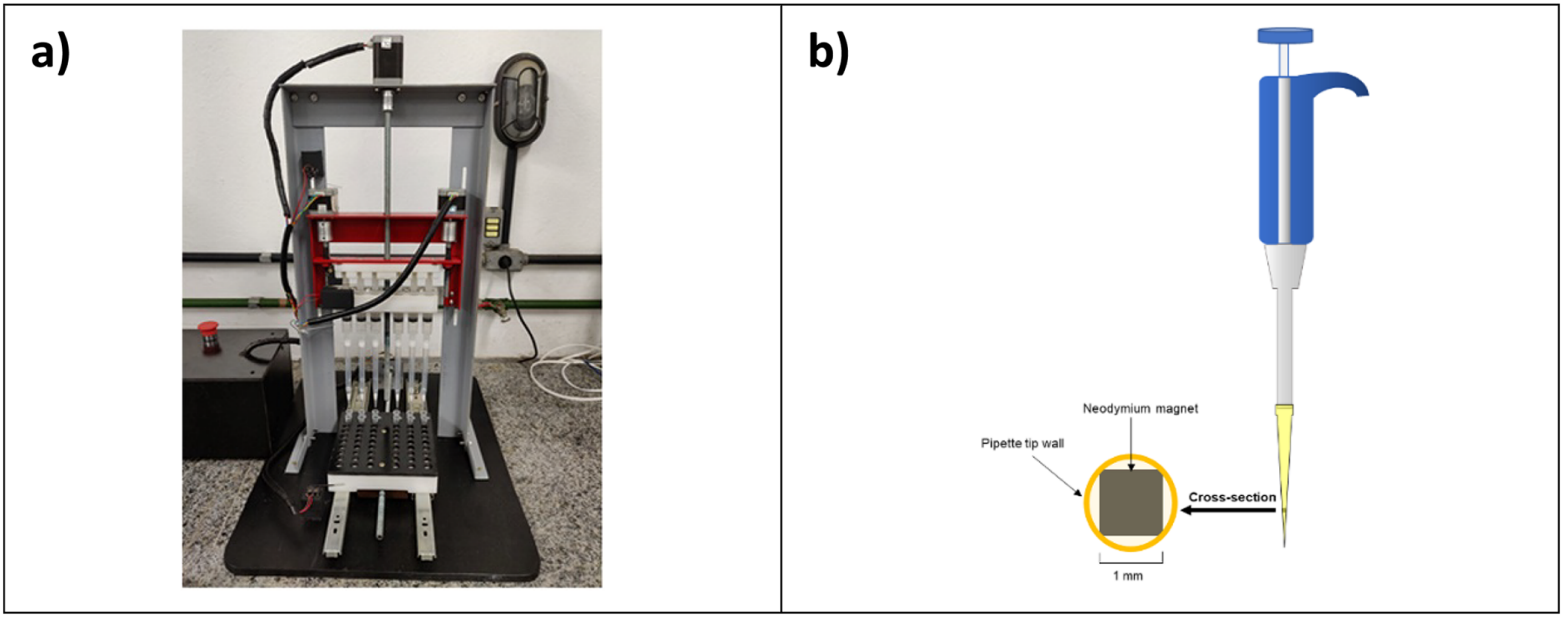
a) The lab-made workstation developed by Carasek’s research group for DPX. Adapted with permission from ref 69. Copyright 2024 Elsevier. b) Proposed device for DMSPE based on pipette tips with neodymium magnets encrusted. Adapted with permission from ref 70. Copyright 2023 Royal Society of Chemistry.

This section should not end without mentioning a smart approach introduced by our research group to conduct DMSPE that was not automated due to lack of resources, but it presents a high potential and ease of automation using pipetting platforms. It consists of conventional pipette tips with neodymium magnets encrusted^70^ (see Figure 5b). The operation starts by pipetting a sample aliquot and ejecting it abruptly over a suspension of a magnetic sorbent contained in a microcentrifuge tube, and then the resulting dispersion is aspirated into the tip in such a way that the magnetic sorbent entrapping the analytes is retained by the encrusted magnet. Then, the sample is discarded, and finally, a small amount of desorption solvent (or washing solvent if needed) is aspirated and dispensed to desorb the analytes, thus providing an extract ready to be measured.

## Automation of Solvent-Based Microextraction Techniques

### Single-Drop Microextraction

Although originally this technique used a water-immiscible organic droplet hanging from the end of a Teflon rod immersed in a vial containing an aqueous sample,^71^ it was rapidly popularized by using a microsyringe.^72,73^ Thus, in close analogy to SPME, a droplet of a few microliters (ca. 1–10 μL) of the water-immiscible organic solvent is carefully taken out from the syringe and left suspended from the tip of the needle, being exposed either to the aqueous sample (i.e., DI-SDME) or to the headspace (i.e., HS-SDME). After a defined period of time, the droplet is retracted back into the syringe needle and transferred to the analytical instrument. DI-SDME can also be accomplished in a three-phase system by using an aqueous droplet immersed into an organic layer in contact with the aqueous sample. This provides excellent results for ionizable compounds by conveniently adjusting the pH of both aqueous phases. The extraction process can even be accelerated via electromigration by applying a potential difference between both aqueous phases, which is known as electromembrane extraction (EME), although there are many other configurations.^74^

Due to the operational workflow, its automation can be accomplished easily by means of commercially available autosamplers. To our knowledge, the first proposal of automated SDME came almost simultaneously from Kokosa’s^75^ and Pawliszyn’s research groups,^76^ who used a CombiPAL autosampler to carry out both the SDME and the subsequent injection into a GC instrument sample by sample. Different articles using this approach, either using the same or similar commercially available autosamplers, were published later. It is worthy to mention the contributions from Hankemeier’s research group, who employed an Advion NanoMate TriVersa robot to conduct EME^77^ directly coupled to a mass spectrometer. In this regard, a 96-well plate was modified by placing a stainless-steel plate in the bottom that acted as the anode, whereas a conductive polymer-made pipette tip acted as a cathode. Even though a 96-well plate was employed, the robot could only perform the extraction of one sample at the same time. In this regard, the samples and the organic solvent for the intermediate layer were placed manually in a 96-well plate. Then, the robot aspirated the desired volume of drop from a selected well and moved to perform the extraction by exposing the drop. After the extraction, the acceptor phase was retrieved, the pipette tip removed from the extraction well, and its vertical orientation changed to a horizontal one before its automatic transference to the mass spectrometer. Similarly, this same research group later proposed a similar workflow using a CombiPAL autosampler to conduct the EME, while the extract was injected directly into an LC instrument.^78^

Leaving aside commercially available expensive solutions, it should be mentioned the lab-made proposal from Santos-Neto’s research group. They employed their already described lab-made Cartesian robotic system^38^ to automate SDME.^79^ In this sense, to perform the SDME, the vials containing the samples and a magnetic stir bar were placed on the magnetic stirrer. The syringe module of the robotic system loaded the extraction solvent from a vial placed in the horizontal platform and then exposed the drop in the sample to perform the extraction of the analytes. After a defined period of time, the drop was transferred to the LC instrument by means of the three-way solenoid valve. While the chromatographic analysis was carried out, the robotic workstation was able to clean the system and prepare the following extraction. Later, these same authors expanded this approach to large drop volumes,^80,81^ although the extract was not transferred directly to the instrument but collected in a tube for an evaporation step prior to its measurement.

Finally, although it was not automated, the proposal resulting from a fruitful collaboration between Carasek’s and Anderson’s research groups to conduct SDME in a 96-well plate format deserves to be mentioned due to its great potential for automation.^82^ These authors fabricated a lab-made extraction device comprised of neodymium magnet rods embedded into inverted pipette tips fixed to pins conveniently disposed for matching into a 96-well plate (see Figure 6). To carry out the extraction procedure, this extraction plate was put in contact with a certain amount of a magnetic ionic liquid previously added to each well of a 96-well plate; thus, it was attracted and kept in each one of the magnet rods due to the magnetic field. Then, these blades were immersed in the vials containing the samples, and after a defined amount of time under stirring conditions, the blades were withdrawn, and the sample plate was manually replaced by a plate filled with a certain amount of the solvent to dissolve the droplet and the extracted analytes. The vertical movement to immerse/remove the extraction device from the 96-well plate was performed manually using a lever.

**Figure 6:**
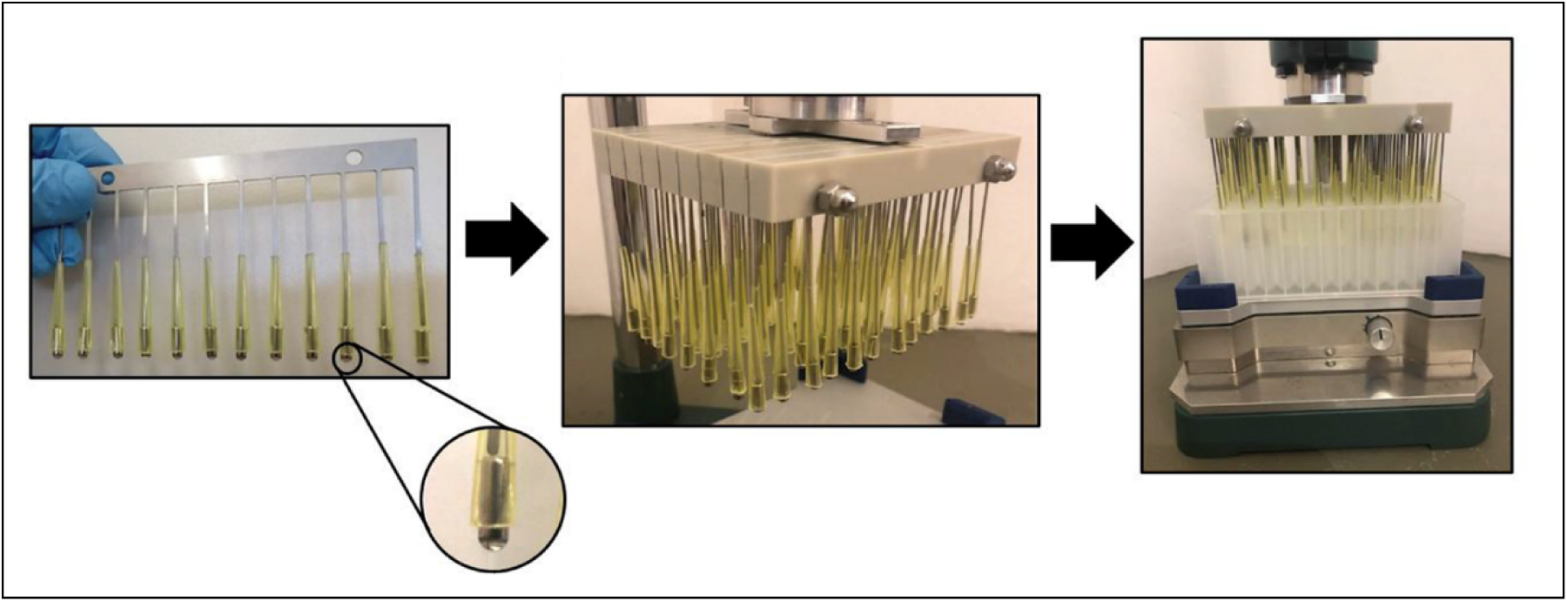
Lab-made extraction device developed by Carasek and coworkers for carrying out parallel SDME. Adapted with permission from ref 82. Copyright 2019 Elsevier.

### Hollow-Fiber Liquid-Phase Microextraction

In 1999, Pedersen-Bjergaard and Rasmussen presented the hollow-fiber liquid-phase microextraction (HF-LPME) approach,^83^ which consisted of a U-shaped porous hollow fiber filled with the extraction solvent immersed in the sample. Both ends of the hollow fiber were inserted into two different syringe needles, one of them to insert the acceptor phase and the other one to collect the acceptor phase after extraction was carried out. A stir bar was placed inside the vial to agitate the sample during the extraction procedure. Some years later, Lee and coworkers replaced the U-shaped fibers with linear shorter ones,^84^ mimicking the SPME.

It should be emphasized that HF-LPME can be carried out under two basic possible configurations, i.e., two-phase and three-phase systems. In the former, a hollow fiber of certain dimensions is clamped to a syringe needle filled with the extraction organic solvent. Then, it is soaked in the same organic solvent, which is immobilized in the pores of the fiber, thus forming the so-called supported liquid membrane. Then, the fiber lumen is filled with the aid of the syringe, and finally, it is exposed to the sample, where target compounds pass through passive diffusion. The three-phase system is similar, but the extraction phase is an aqueous solution conveniently pH-adjusted in such a way that the organic supported liquid membrane avoids the mixing between both donor and acceptor aqueous phases. This three-phase system is used for the extraction of potentially ionizable compounds, which pass from the aqueous donor solution to the supported liquid membrane, and then they are back-extracted into the acceptor aqueous solution located at the lumen of the hollow fiber. In both cases, the acceptor phases are withdrawn with the syringe after extraction and transferred to the analytical instrument.

Pedersen-Bjergaard and Rasmussen^85^ also proposed in 2006 the application of an electric field as a driving force for the electrokinetic migration of charged solutes between two aqueous compartments throughout a supported liquid membrane, which was coined as EME, as mentioned previously.

As in SDME discussed earlier, its operational workflow allows HF-LPME to be easily automated by using commercially available liquid handlers. As far as we know, the first attempt to automate HF-LPME is that reported by Zhao and Lee,^86^ who employed a programmable syringe pump for compressing/pulling the plunger of a microsyringe, thus conducting dynamic HF-LPME. Initially, a predefined volume of extraction solvent was drawn into the syringe, which was then inserted into the hollow fiber. Then, the fiber was soaked in a vial with more extraction solvent to fill the pores, and then it was removed, and the solvent from the syringe was ejected to fill the fiber lumen. After that, the syringe was fixed on the syringe pump holder, and the fiber was exposed to the sample (placed on a magnetic stirrer) (see Figure 7a). All the described steps so far were made manually, but then, the plunger of the syringe was withdrawn, thus entering the sample into the hollow fiber and retrieving the extraction solvent inside the needle of the syringe. Then, the opposite procedure was performed, ejecting the sample inside the fiber and placing the extraction solvent on it. The procedure was repeated as many times as needed. Finally, the magnetic stirrer and the syringe pump were switched off, and the syringe was manually removed from the sample solution, thus injecting the extract into the analytical instrument.

**Figure 7:**
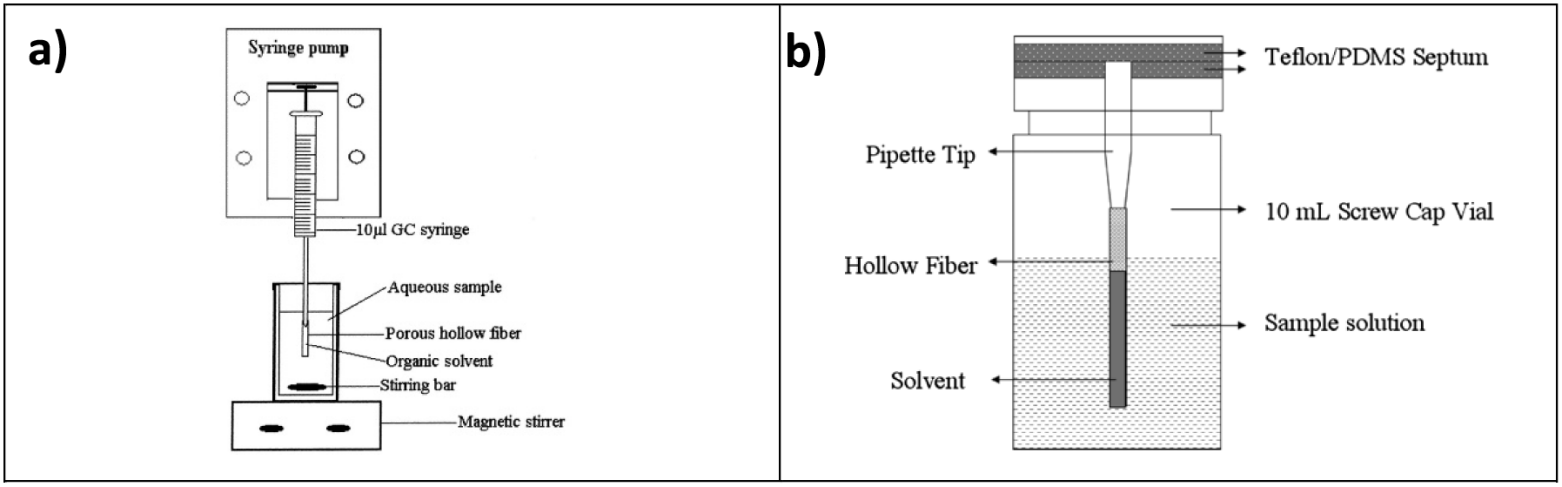
a) Semiautomated system to perform dynamic HF-LPME proposed by Zhao and Lee. Adapted with permission from ref 86. Copyright 2002 American Chemical Society. b) Extraction vial designed by Ouyang and Pawliszyn for automated HF-LPME. Reproduced with permission from ref 87. Copyright 2006 American Chemical Society.

Ouyang and Pawliszyn also used a commercially available solution to contribute to the automation of HF-LPME. They employed a CombiPAL system to handle a microsyringe, but in this case, the hollow fiber was not clamped to the syringe needle but to a pipette tip, which was fixed in the septum of a vial^87^ (see Figure 7b). In this sense, all vials containing the hollow fibers were placed on the tray of the autosampler. Then, the fiber was filled with the extraction solvent, and the vial was transferred to the vortex agitator with a temperature controller. After extraction, the vial was returned to the sample tray, and the extraction solvent was collected from the hollow fiber and injected automatically into the analytical instrument.

Here again it should be cited the lab-made robotic workstation^38^ developed by Santos-Neto’s research group, who adapted it for carrying out HF-LPME.^88^ The clamping of the hollow fiber to the syringe was made manually, and then the fiber was automatically filled with the extraction solvent by the autosampler to conduct the extraction procedure. Once the extraction was finished, the extract was automatically transferred to the LC system through the three-way solenoid valve.

It should be mentioned here the proposal from Li and Bao,^89^ who devised a platform to conduct HF-LPME in parallel, based on the concept of a 96-well plate. Similar to the extraction vial designed by Ouyang and Paliwszyn (see Figure 7b), these authors constructed a plastic cover with 96 holes attached directly to one open end of 96 hollow fibers using glue, while the other end of the hollow fibers was sealed. The plastic cover with 96 hollow fibers completely matched a commercial 96-well plate. Each hollow fiber was manually filled with the acceptor phase using a pipette, and the entire platform was fitted into a 96-well plate containing the samples, and it was orbitally shaken. After extraction, the extracts were taken manually by a pipette and transferred for analysis. It is true that it was not an automated workstation, but it presents a high potential for this by using a liquid handling workstation.

Carasek and coworkers also applied their above-described lab-made proposal (see Figures 3b and 5) for hollow-fiber renewal liquid membrane extraction, a modality of HF-LPME where a small amount of extraction solvent is added to the sample to provide a high organic/aqueous ratio with constant renewal of the liquid surface of the supported liquid membrane.^90^ In this case, they manually inserted 1.0 cm-length hollow fibers into an 8 × 12 pins matrix to get the extraction device. First, the extraction device was soaked in a 96-well plate filled with the appropriate organic solvent to create the supported liquid membrane on the pores of the hollow fibers. Then, the extraction device was raised, the 96-well plate was changed by another one filled with the samples to perform the extraction step, and finally, the desorption step was carried out by changing to another 96-well plate with the desorption solvent. Orbital stirring was used in each step, and the up-and-down movement of the extraction device was performed manually by means of a lever, as described previously. Later, this same research group, in a study led by Merib, improved their workstation by automating the up-and-down movement of the extraction device by means of a stepper motor controlled by Arduino, similar to their workstation described before for PANDA (see Figure 3c). The operational workflow can be envisioned at https://www.sciencedirect.com/science/article/pii/S0021967320303095?via%253Dihub#ecom0002.

Aware of the difficulty to implement a multiwell format in HF-LPME and, consequently, its subsequent automation, Pedersen-Bjergaard and Rasmussen, i.e., the inventors of the HF-LPME themselves, developed a new microextraction approach using flat membranes in a 96-well plate sandwich format coined as parallel artificial liquid membrane extraction (PALME).^91^ The extraction device consists of a conventional 96-well plate containing the samples and a 96-well filter plate originally intended for filtration where the membranes are impregnated with an organic solvent to form the supported liquid membrane. Both plates are manually fitted, and the acceptor solution is added to the supported liquid membrane. After orbital stirring, the extracts were transferred to the analytical instrument. Despite not being automated, its potential for automation by using a pipetting workstation with a 96-well plate-handling arm is undeniable.

### Dispersive Liquid–Liquid Microextraction

Dispersive liquid–liquid microextraction (DLLME), proposed by Rezaee et al. in 2006,^92^ is by far the most popular solvent-based microextraction technique due to its simplicity and rapidity. The original workflow is based on a solvent ternary system, i.e., a water-immiscible extraction organic solvent is mixed with a water-miscible organic solvent (disperser solvent), and subsequently, this mixture is rapidly injected with the aid of a syringe into the aqueous sample contained in a conical tip tube. The disperser solvent causes the total dispersion of the water-immiscible extraction solvent within the aqueous donor phase, forming what is known as a cloudy solution, where the extraction solvent forms thousands of fine droplets. This makes the contact area between the extraction solvent and the sample extremely large, so the equilibrium state is quickly achieved. Then, phase separation is accomplished by centrifugation, and the extract is retrieved with the aid of a syringe and transferred to an analytical instrument. Usually, extraction solvents denser than water are preferred, which facilitates its retrieval from the conical tip, where it forms a drop, rather than from the top, where it forms a very thin layer. Nevertheless, different strategies have been proposed, with the most popular being the freezing of the extraction solvent, which causes the solvent to solidify into a droplet shape, or the use of dedicated extraction devices that allow the aqueous phase to be drained from the bottom, or adding water to raise the liquid level to a narrow neck, etc.^93^

Due to its operational workflow, including centrifugation for phase separation, the automation of DLLME is not as easy as other microextraction approaches. In fact, it was not until 2014 that the first contribution on automated DLLME appeared, by Lee and Guo, who employed a demulsification solvent to avoid centrifugation and custom-made narrow-necked vials from their glassblowing workshop to facilitate the extract collection^94^ (see Figure 8a). For this purpose, the sample vials, the demulsification agent, and a vial with a mixture of the extraction and the disperser solvents were placed in a CombiPAL robotic workstation tray. Then, the autosampler filled the syringe with the corresponding volume of the extraction/disperser solvents mixture and injected it into the sample vial to form the cloudy solution. After that, the autosampler injected a predefined volume of demulsification agent to break up the emulsion and to raise the organic phase up to the narrow neck, from where it was collected and injected into the analytical instrument. The use of demulsifiers to break up the emulsion, thus avoiding the centrifugation step, was also subsequently exploited by Jing’s research group by using an 8-channel automated pipetting workstation to prepare four samples simultaneously over 24-well plates.^95^ The 8-channel arm allowed an effective mixing of the sample and both disperser and extraction solvents by aspirating/dispensing the mixture several times.

**Figure 8:**
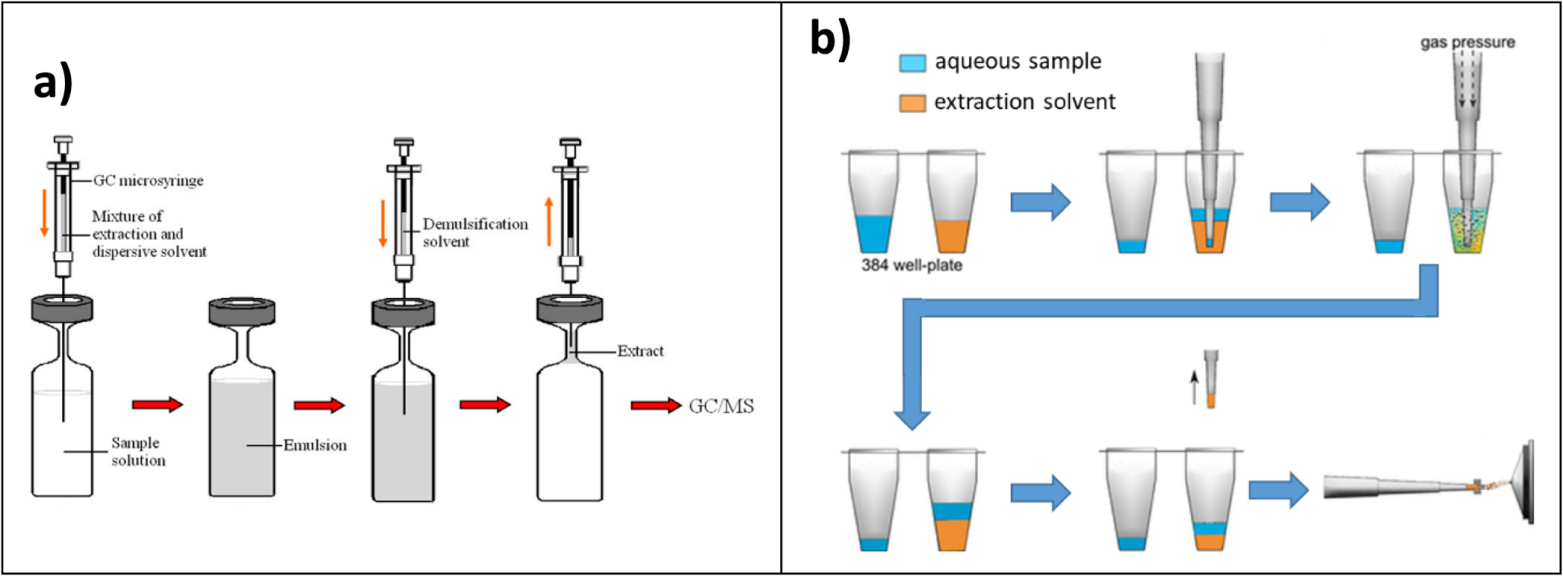
a) Custom-made narrow-necked vials and operational workflow carried out by Guo and Lee for automated DLLME. Adapted with permission from ref 94. Copyright 2014 American Chemical Society. b) Schematic illustration of the operational workflow conducted by Hankemeier’s research group for automated DLLME. Adapted with permission from ref 96. Copyright 2014 American Chemical Society.

Almost simultaneously, Hankemeier’s research group proposed another automated DLLME strategy using a Triversa Nanomet commercially available robot.^96^ The unique manual step was the prefilling of the 384-well plate. Even though this multiposition platform was employed, the extraction of only one sample at the same time was performed. After prefilling the plate (see Figure 8b), the sample was taken and dispensed into a well containing the extraction solvent. Then, both phases were mixed bubbling nitrogen during a period of time. Subsequently, both phases were allowed to separate naturally, and finally an aliquot of the extract was automatically transferred to the mass spectrometer.

## Discussion

### Throughput

As mentioned earlier, in general, automated systems can perform operational tasks faster than humans, which results in the throughput, understood as the number of samples processed within a given period of time. In high-demand environments, throughput is a critical feature that directly influences operational costs, turnaround times, and overall laboratory efficiency. This section presents a general comparison of the throughput achieved by using automated systems to that of manual procedures.

First, it should be mentioned that, leaving aside sample pretreatment operations (e.g., filtration, centrifugation, decantation, etc.), an analytical procedure involving (micro)­extraction techniques can be divided into two major steps, namely, the extraction itself and the instrumental measurement. Thus, the throughput of the entire analytical procedure is determined by the throughput of both these steps. In the case of the extraction, the throughput can be improved by reducing the total time of this step or performing parallel extractions. Regarding the instrumental measurement, the only way to increase the throughput is by requiring a short-time analysis, with techniques having instantaneous response (e.g., sensors) being an ideal case. In this regard, two scenarios should be distinguished depending on whether extraction or measurement is the limiting step.

Being aware that there are many microextraction techniques, which operate under very different workflows, with different levels of automation within each, and allowing either the processing of just one sample (i.e., single) or several samples simultaneously (i.e., multi), typically using standardized 48-, 96-, or 384-well plates, one might imagine that making a comprehensive comparison is hardly achievable. Thus, with the purpose of discussing and making the reader reflect on the benefits of automated procedures in terms of throughput, it has been deemed appropriate to choose SPME with thermal desorption into a GC as an example. Figure 9 graphically compares, in a qualitative and relative way, the total time estimated to conduct the SPME-thermal desorption-GC analysis of a hypothetical group of analytes in three samples, if the procedure is either manual or automated (e.g., using a CombiPAL robot), if single/multiextraction/s are performed, or if extraction/instrumental measurement is the limiting step. The duration of the instrumental measurement is considered the same for both automated and manual procedures to obtain a fair comparison. Moreover, it has been considered that both manual and automated systems are capable of extracting a sample while the previous one is being measured in the corresponding instrument. Nevertheless, the time extension considered in each step is only indicative, and it is intended to illustrate, just as an example, the time spent in each step for a better understanding. However, real and concrete data from both the extraction and instrumental measurement steps must be used when comparing specific methods in a more exhaustive way.

**Figure 9:**
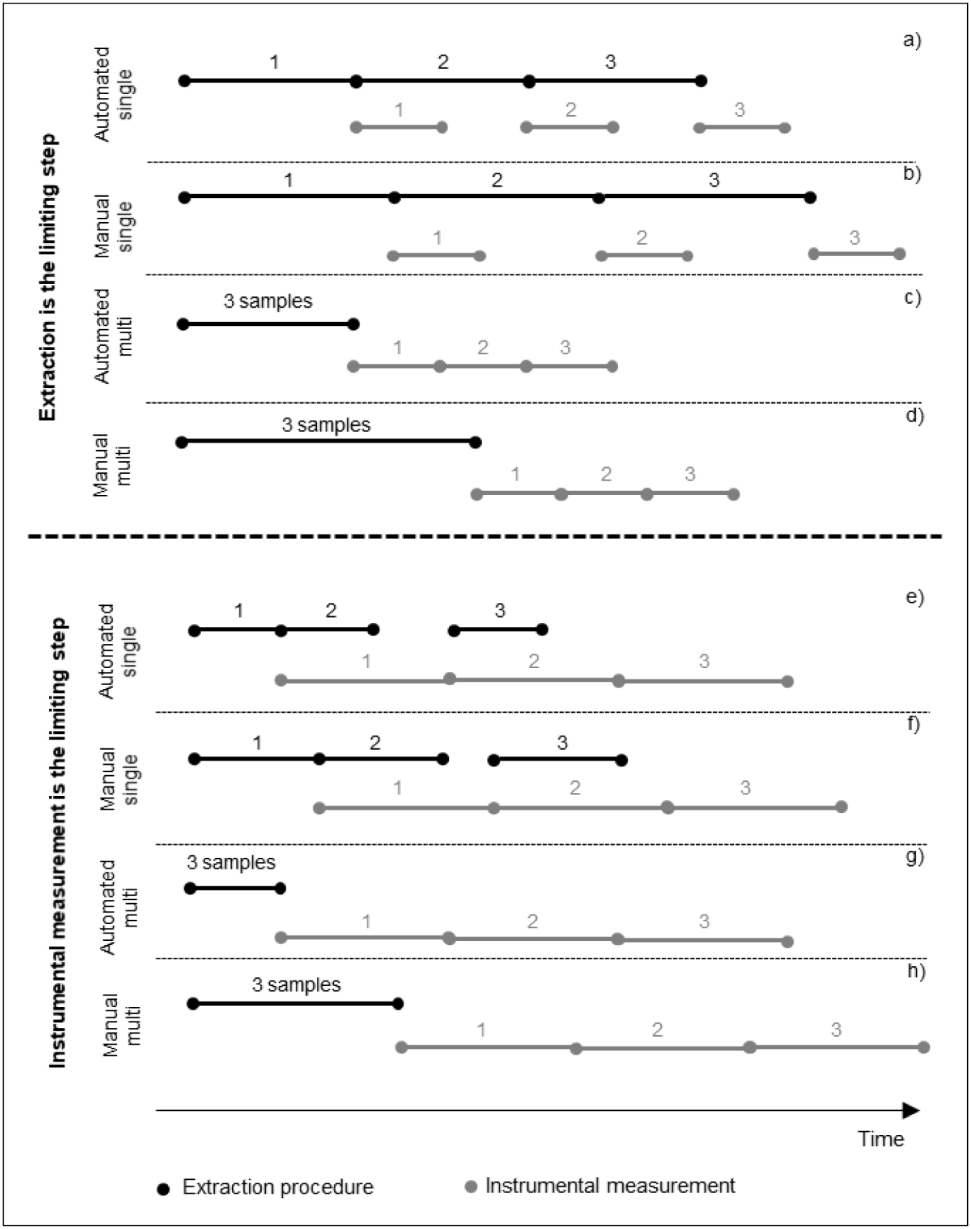
Comparison in terms of throughput between manual and automated procedures depending on the limiting step and the single or multiextraction configuration for the treatment of three samples. The numbers (1, 2, 3) represent the number of the sample treated.

In the particular case of single extraction and when extraction is the limiting step, higher throughput is obtained for the automated method (Figure 9a) rather than the manual method (Figure 9b), as the former allows for quicker performance of the operational tasks, e.g., placing the fiber in the sample solution to extract the analytes and then moving the fiber to the GC injection port, and, unlike manual workflow, the fiber does not need to be clamped/released every time. The more samples processed, the greater the time difference between automated and manual procedures for analyzing the same number of samples (i.e., the time difference at the end of the instrumental measurement of sample 2 is greater than at the end of sample 1, and so on). Therefore, the more samples that are processed, the greater the benefit of automation.

In the case of single extraction, when the limiting step is the instrumental measurement (Figure 9e and f), the overall throughput difference is less significant, since the extraction of successive samples is being performed during the measurement of the previous one, and thus, the overall difference is just the difference in the time required for the first extraction. Note that sample 3, and successive ones (if any), cannot be extracted until sample 2 is injected, since the extraction device (i.e., SPME fiber) is needed, and that is why there is a gap.

For multiextraction systems, i.e., parallel extractions, it should be said that this option is not as recurrent in manual systems as it is in automated ones. Nevertheless, in this case, a priori, it could be erroneously assumed that the difference between automated (Figure 9c) and manual (Figure 9d) configurations is less than in the single extraction system discussed above. However, whereas a multichannel automated arm can perform the three extractions simultaneously using the same amount of time as for one extraction (compare Figure 9a and c), in the manual way, the extraction device needs to be prepared (clamp/release) thrice, and thus the time for the extraction step is accordingly lengthened in the comparing time scale, and thus the difference is even higher than in single extraction systems. The more samples processed, the greater the difference and thus the benefit of automation.

This is also observed when the limiting step is the instrumental measurement (see Figure 9g and h). It should be taken into account that all of the samples are submitted to the measurement step when all of them have been extracted, and the measuring instrument (i.e., GC in this case) cannot measure all of the extracted samples simultaneously but sequentially.

Finally, the most usual situation is compared, which means an automated multiextraction system versus a manual single-extraction system. In this case, the automated systems demonstrate higher throughput because they combine the two previously mentioned benefits: faster operation and the ability to perform multiple extractions simultaneously. When extraction is the limiting step, the throughput difference between automated multiextraction (Figure 9c) and manual single (see Figure 9b) is substantial, particularly when processing a large number of samples. The greater the number of samples treated, the greater the advantage provided by automated multiextraction systems. However, when the limiting step is the instrumental measurement, a smaller difference in throughput is obtained (Figure 9g and f). In this case, the only difference lies in the time spent extracting the first sample in the manual system and extracting the various samples using the automated system, since the rest of the samples extracted manually are being extracted while the previous ones are being measured. Nonetheless, the presence of an analyst is continually needed in the manual procedure, whereas in the automated one, it is just at first to program the sequence.

It should be emphasized that these differences depend on the microextraction technique used, as the degree of manipulation required varies among them. Thus, automation offers greater advantages when using microextraction techniques that involve a high degree of manipulation.

### Degree of Automation

As described above, a high degree of automation, which therefore reduces manual intervention, offers significant advantages. Nevertheless, even if the extraction stage is fully automated, manual intervention is a prerequisite to transfer the sample(s) to the extraction device or to place vials containing samples into the extraction system. Additionally, some other manual operations may still be required for adding/removing dedicated solvents or to transfer the extract to the measuring instrument, depending on the degree of automation achieved. In this sense, for a system to be considered fully automated, it must perform the entire analytical procedure without requiring human intervention beyond the initial loading of samples into the extraction system and programming of the sequence. Thus, the ideal case would be a fully automated extraction platform integrated with the measuring instrument that can carry out the extraction and then transfer the extract to the instrument itself. These systems can process a high number of samples without human intervention, offering the advantage of operating continuously 24 h a day. This means that a large number of samples can be processed and measured daily, even if the entire system itself does not have a high throughput.

The degree of human intervention and the ability to perform sample extraction and their subsequent measurement were already discussed for each of the robotic workstations described above. Nevertheless, it should be emphasized that the high degree of automation achieved by using CombiPAL (and similar) robotic workstations directly mounted on GC or LC chromatographic systems, which, although one by one, perform all the needed steps for the extraction and transfer to the injection port of the measuring instrument based on a syringe workflow. These systems have been employed to perform MEPS,^36^ SPME,^47^ DMSPE,^60^ SDME,^75,76,78^ HF-LPME,^87^ and DLLME.^94^

Another fully automated method was accomplished by Hankemeier’s research group for EME by using an Advion NanoMate TriVersa robot,^77^ which uses a head with a pipette tip instead of a syringe like CombiPAL. After the extraction, the vertical orientation of the head changed to a horizontal one, and it was directly infused into the mass spectrometer by electrospray ionization.

Other commercially available solutions with a lower degree of automation were already described. Most of them require human intervention for transferring the extract to the analytical instrument. In this regard, it should be mentioned the specific case of liquid-handling robotic workstations, which conduct μSPE rapidly by aspirating/withdrawing the dedicated solvents into 96-well plates subjected to the extraction in parallel, but they require the manual transfer of the extraction plate to the analytical instrument. At the opposite end, it is worth mentioning the contributions of Santos-Neto’s research group employing a lab-made robotic workstation to carry out MEPS,^39^ SPME,^55^ SDME,^79^ and HF-LPME.^88^ These authors used a three-way solenoid valve positioned in the head of the syringe-based extraction device in such a way that the syringe was connected to the extraction vial or to the LC injection loop^38^ (Figure 10). Thus, after extraction, the extract was directly transferred to the analytical instrument without human intervention.

**Figure 10:**
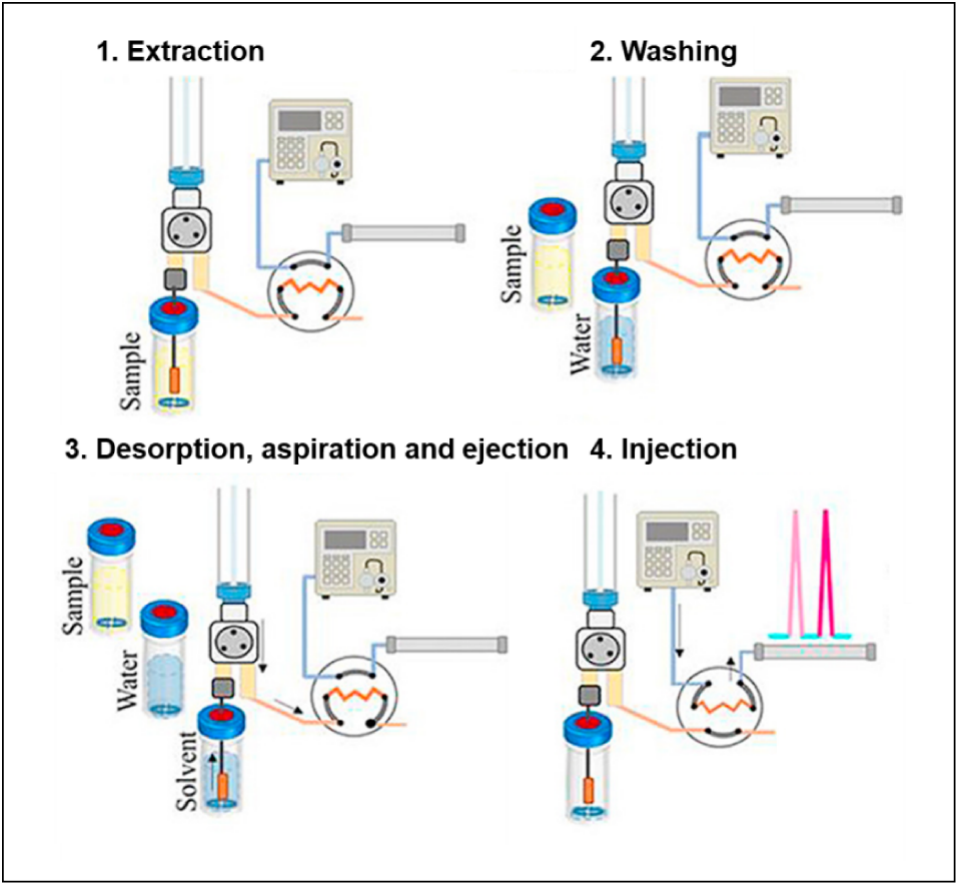
Automated workflow achieved by the lab-made robotic workstation developed by Santos-Neto’s research group for transferring the extract to the analytical instrument. Adapted with permission from ref 55. Copyright 2021 Elsevier.

It should also be noted that some microextraction techniques need human intervention to conduct operations inherently associated with the technique itself, such as HF-LPME, which requires the manual removal of the used hollow fiber in the previous extraction and the clamping of the new one into the syringe needle for the extraction of the following sample. In this regard, it is worth emphasizing the contribution of Ouyang and Pawliszyn.^87^ These authors designed *ad hoc* vials, where the hollow fiber was clamped to a precut pipette tip fixed to the septum of the vial, acting as a needle guide for the CombiPAL autosampler, thus avoiding the manual intervention.

Finally, there are workstations that require the presence of the operator during the extraction procedure, and it is also necessary to manually transfer the extract to the measuring instrument, as it is the case of the ones to carry out DMSPE^62^ or HF-LPME.^86^ These workstations have the lowest degree of automation and, therefore, do not offer all the benefits that automation provides.

### Portability and Economic Considerations

One feature that has gained attention in recent years is the portability of analytical instrumentation. This is because both the transport and storage of the samples are critical stages, and if they are not carried out correctly, alterations in their composition can occur, potentially degrading the compounds of interest. Furthermore, these two stages are associated with a greater consumption of time and economic resources. Thus, there is an increasing demand for methodologies where the extraction procedure is carried out on-site and, if possible, the instrumental measurement too. In the case of the described automated approaches, complex and large commercially available workstations (e.g., liquid-handling workstations) were employed, making their portability very difficult due to the size and the energy demand.

Being aware of this situation, different lab-made solutions have been proposed. It should be mentioned that the lab-made robotic workstation was developed to carry out DMSPE.^62^ In this workstation, all the electronic gadgets are placed inside a 3D-printed casing with dimensions of 20.2 cm length × 19.4 cm width × 10.2 cm height, allowing for its total portability. Furthermore, it can be powered by an external battery of 9 V, without the need for a direct plug into an electrical network.

Another relevant aspect when developing automated systems is the economic cost of the equipment. Thus, commercially available robotic workstations are only affordable for high-budget laboratories, as their price is very elevated. In this respect, alternative systems based on open-source software and assembled with low-cost gadgets have been proposed, ensuring the affordability of the system. It should be mentioned the proposals from Santos-Neto’s research group to carry out MEPS,^39^ SPME,^55^ SDME,^79^ and HF-LPME;^88^ those from Carasek and Merib to carry out SPME^57^ and HF-LPME;^90^ and that from Chisvert’s research group to carry out DMSPE.^62^ All of them were developed employing low-cost gadgets and controlled by the open-source software Arduino. Furthermore, these systems can be modified and upgraded easily depending on the requirements of the method (e.g., adding new features).

Regarding economic benefits, several aspects should be considered when comparing automated methods with manual procedures. On the one hand, as mentioned before, the throughput of the method: the more samples that can be processed within a given period of time, the greater the economic benefits. On the other hand, the degree of automation of the extraction procedure, the automated analysis of subsequent samples, and the online instrumental measurement should also be taken into account. If a method incorporates these three features, operator intervention is greatly reduced, as is the number of personnel required, thereby lowering overall operating costs. If the extraction procedure is automated or only easy manual steps are required, then less qualified operators can carry out the procedure. As it has been described above, the intervention of the operator is different for each approach. In this sense, the approaches where no human intervention is required (except for the placement of the samples in the system) will be safer for the operator, as the manipulation of the reagents is minimized.

## Concluding Remarks and Future Perspectives

As has been demonstrated, the automation of the sample preparation stage has been an important focus in the field of analytical chemistry in recent decades. In this regard, various robotic workstations have been developed to perform the most widely used microextraction techniques, offering advantages over those of manual procedures. While automation using commercial robotic workstations has been widely developed, automation based on lab-made robotic workstations has been accomplished to a much lesser extent. Even more, it should be mentioned that it represents a challenging task due to the high variability of the involved operational workflows, which means that some techniques are easier to automate than others depending on their specific experimental workflow. In this regard, those that can be performed using autosamplers or liquid handlers, such as SPME or DPX, are easier to automate than those requiring additional instrumentation, such as DMSPE or DLLME, which require retrieving the sorbent or solvent, respectively, after its dispersion in the sample solution. It should also be pointed out that some of the lab-made proposals are only partially automated, and there exist other interesting approaches that are not automated at all despite their high potential for it. All this is most probably due to the lack of economic resources.

As discussed, automated systems generally offer higher productivity compared with manual ones. Furthermore, it is crucial to develop systems that enable the integration of the microextraction system into the measuring instrument, thus allowing the online measurement subsequent to the microextraction step. Another important aspect is the degree of automation of the analytical procedures. In this regard, it is crucial to develop fully automated analytical procedures that do not require manual intervention. The only human intervention would be placing the samples and reagents in their designated locations at the beginning of the analytical procedure. This would lead to less human intervention, resulting in safer procedures and lower cost. Despite the benefits of robotic systems, one of their biggest limitations is their portability because of their large size.

In this regard, although considerable progress has been achieved, further research is still necessary, and future perspectives in the field are likely to focus on four key aspects. First, increasing sample throughput through workstations capable of performing parallel extractions, regardless of the microextraction approach, although kinetically favored approaches such as dispersive ones, are promising candidates. Second, enhance automation by minimizing or eliminating manual operations like solvent addition/removal or extract transfer to the measurement instrument. Third, minimalist and compact workstations should be designed to improve portability and facilitate on-site applications. Finally, future efforts should also address the fabrication of these workstations using low-cost electronic devices controlled by open-source software. Overall, continued research is essential to achieve the ideal robotic workstation for microextraction, i.e., a portable dispersive-based multiextraction device requiring minimal human intervention, fabricated using low-cost electronic gadgets, and controlled by open-source software.
